# Establishment of a method for the determination of hydrogen sulfide in human serum by liquid chromatography-tandem mass spectrometry and evaluation of its clinical application: Determination of H _2_S using LC-MS/MS

**DOI:** 10.3724/abbs.2024007

**Published:** 2024-02-20

**Authors:** Chunyan Li, Jia Chen, Yu Su, Wuzheng Liu, Xiangyu Meng, Ao Zhang, Yitong Bai, Yingzi Liu, Ruichen Liu, Lin Zhang, Jun Wu

**Affiliations:** 1 Beijing Jishuitan Hospital Capital Medical University Beijing 100035 China; 2 Department of Laboratory Medicine Peking University Fourth School of Clinical Medicine Beijing Jishuitan Hospital Beijing 100035 China; 3 Peking University Beijing 100000 China; 4 Shanghai AB Sciex Analytical Instrument Trading Co. Ltd. Shanghai 100027 China; 5 Department of Geriatric Cardiology the Second Medical Centre Chinese PLA General Hospital Beijing 100853 China

Hydrogen sulfide (H _2_S) is an inorganic compound. It is a colorless, water-soluble gas that results in the smell of rotten eggs. Hydrogen sulfide is the third most important endogenous gas signaling molecule after nitric oxide, NO, and carbon monoxide (CO) [1]. Hydrogen sulfide is involved in many physiological and pathological processes. Relevant studies have shown that hydrogen sulfide is widely distributed in the cardiovascular, nervous, digestive, endocrine, and immune systems. It is involved in many pathophysiological processes [2]. It has been reported that H _2_S is important for regulating bone metabolism and treating bone diseases such as osteoporosis [3]. H _2_S is expected to become a new target for studying bone metabolism and bone-related diseases [3]. Currently, the commonly used detection methods for H _2_S at home and abroad include gas chromatography, the electrode method, and methylene blue spectrophotometry [1]. To date, gas chromatography has been the gold standard for detecting H _2_S in serum [4].

Although gas chromatography has high sensitivity and good specificity [5], the disadvantage of meteorological chromatography is that it has higher requirements for detection, standard gas and sampling technology, and meteorological chromatography equipment. The detection equipment is expensive and has a long detection time [4]. Therefore, this technique is generally not applied in clinical or scientific experiments. In addition, the electrode method destroys the protein structure of the sample through strong alkali conditions, and then the H _2_S content in the sample is determined by detecting S ^2–^ [ 2, 6]. However, because the electrode method has a high demand for sample quantity and poor reproducibility, it has not been widely promoted. The methylene blue spectrophotometric operation is simple [7], the reagent used is relatively inexpensive, the detection time is relatively short, and the required instruments are relatively common; however, the sensitivity of the spectrophotometer is poor, and only more than 10 μM H _2_S can be detected via the spectrophotometer method. Therefore, how to improve the detection method of endogenous H _2_S to improve the accuracy and sensitivity of H _2_S detection is a problem that needs to be solved.

High-performance liquid chromatography-tandem mass spectrometry (LC-MS/MS) has been widely used in clinical testing in recent years due to its high specificity and sensitivity, wide linear range and high stability [ 8, 9]. This method has the advantages of good linearity and high accuracy, and the analysis of each sample index requires only a few minutes.

First, the C18 column and T3 column were tested according to conventional methods [9], and it was found that the methylene blue chromatographic peaks had different degrees of trailing and that the peak width was greater. The amino column was subsequently replaced, but the methylene blue chromatographic peak still exhibited a small range of trailing. A sharp symmetric peak shape was obtained by adding ammonium formate to adjust the ionic strength of the mobile phase.

Due to the low content of H _2_S in blood, zinc sulfide precipitates generated by the H _2_S reaction in blood samples are almost invisible, and there is a high probability that zinc sulfide precipitates will be removed by cleaning, leading to problems in standard curves and reproducibility. Therefore, the 1% zinc acetate solution was replaced by a zinc hydroxide suspension ( *i* . *e*., 1% zinc acetate solution: 1.5 M sodium hydroxide solution, V:V=7:1) to increase the precipitation such that zinc sulfide could attach to the zinc hydroxide particles and avoid being absorbed by the pipette gun.

The 10 mM sodium sulfide stock solution (10 μL) was accurately diluted to 100 μM, which is the highest point of the standard curve S1. Then, S1 was diluted to 50 μM, which is the secondary peak at S2 in the standard curve. After the above steps were used to dilute S8, a sodium sulfide standard curve was constructed, and the concentrations of S1‒S8 were 100 μM, 50 μM, 25 μM, 12.5 μM, 6.25 μM, 3.13 μM, 1.56 μM, and 0.78 μM, respectively.

The chromatogram was collected after the reaction of the standard curve. The concentration of methylene blue (sodium sulfide) was taken as the horizontal coordinate (X), the response peak area of methylene blue was taken as the vertical coordinate (Y), and the weight was 1/X. The standard curve equation for H _2_S was Y=30.208678X+4938.01, R2=0.9985 ( Figure 1), and the accuracy of all points in the standard curve was greater than 80%. The quantitative detection requirements were met. The lower limit of standard curve quantitation was 0.78 μM, and the signal-to-noise ratio was S/N>100.

**Figure 1:**
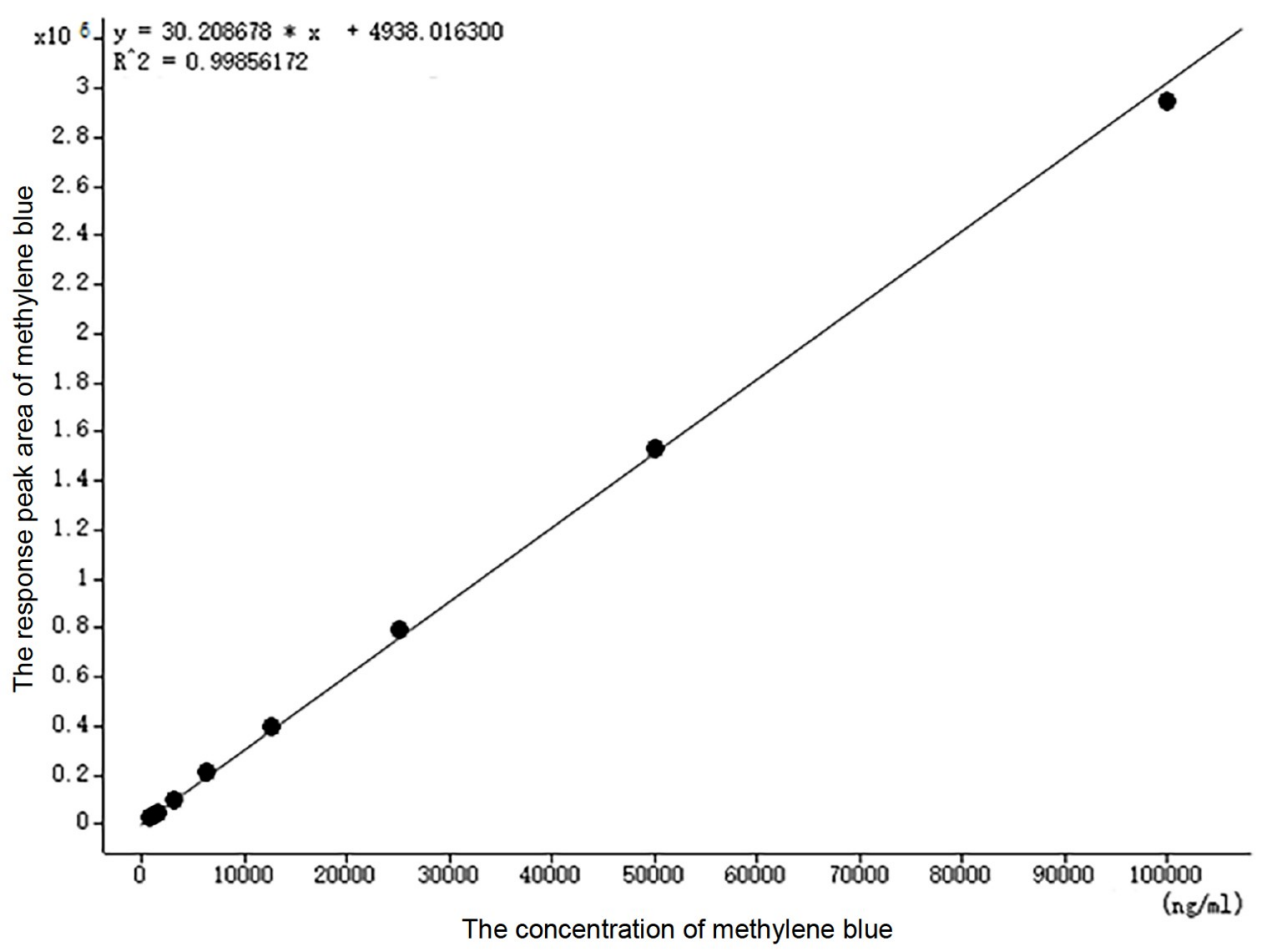
Figure 1 Standard curve

The samples were thawed in an ice bath followed by vortex mixing. To 50 μL of the sample, 100 μL of a 1.5 M sodium hydroxide solution was added and vortex mixed. Subsequently, 300 μL of the extraction reagent (1% zinc acetate solution: 1.5 M sodium hydroxide solution, V:V=7:1, Turbidity) was added, and the mixture was vortexed. An ultrasonic treatment was then performed in an ice water bath for 5 min, followed by centrifugation at 12,000 *g* at 4°C for 15 min. The supernatant was carefully removed, and 200 μL of pure water was added. Next, 50 μL of the reaction reagent (N,n-dimethyl-p-phenylenediamine:ferric chloride, V:V=1:1, mixed immediately) was added to the solution, which was vortex mixed. An additional ultrasonic treatment in an ice water bath for 5 min was carried out. Then, 250 μL of methanol was added, and the mixture was vortex mixed again. The final step involved centrifugation at 12,000 *g* for 10 min at 4°C. The supernatant obtained (120 μL) was then taken for detection using the designated machine.

The study was approved by the Ethics Committee and validated by the Beijing Jishuitan Hospital, and all participants provided signed informed consent. Serum samples from 129 women with or without postmenopausal osteoporosis were measured. The inclusion criterion for osteoporotic women was postmenopausal status and vertebral compression fracture under nonvolatile conditions. The exclusion criteria were patients with liver disease, renal disease, or diabetes mellitus. The inclusion criterion for the control group was no women. All the serum samples were obtained in tubes with a clot activator (Vacuette; Greiner Bio-Onopausal wne, Essen, Germany), and the tubes were protected from light, centrifuged for 10 min at 3727 *g* and immediately stored at –80°C. The samples were stored for a maximum of 3 months before analysis.

The procedural steps for the sodium sulfide standard curve working liquid reaction are as follows: for each point on the standard curve, a 50 μL aliquot of the solution was extracted. Subsequently, 150 μL of pure water is introduced, and the mixture is subjected to vortex mixing. Following this, 50 μL of the reaction reagent (comprising N,n-dimethyl p-phenylenediamine and Ferric chloride in a volumetric ratio of 1:1, currently in use) was added, and the solution was vortex mixed again. The resulting mixture underwent an ultrasonic soak in an ice water bath for 5 min. After ultrasonication, 250 μL of methanol was added, and the solution was once again subjected to vortex mixing. The subsequent step involved centrifugation at 4°C and 12,000 *g* for 10 min. Following centrifugation, a 120 μL aliquot of the supernatant was carefully collected and subsequently utilized for testing through the designated machine.

The chromatographic column used was an AB SCIEX Triple quad 4500+. Mobile phase A was an aqueous solution containing 0.1% formic acid and 5 mM ammonium formate. Mobile phase B was 90% acetonitrile solution containing 0.1% formic acid and 5 mM ammonium formate. The column temperature was 40°C, the sample temperature was 4°C, and the sample volume was 1 μL. The elution gradient is shown in Table 1.

**Table:** **Table 1** Liquid phase gradient elution condition

Time (min)	A phase (%)	B phase (%)
0.00	5	95
1.50	5	95
4.00	50	50
6.00	50	50
6.50	5	95
10.00	5	95

The ion source used was an electrospray ionization (ESI) source in positive ion detection mode, and a triple quadrupole analyzer was used. The scanning mode was multiple reaction monitoring (MRM). The conditions were as follows: sheath temperature: 300°C; sheath gas flow rate: 6 L/min; dry temperature: 350°C; drying gas flow rate: 10 L/min; capillary voltage: 4000 V; cone-hole voltage: 500 V; methylene blue parent ion: 284.1 m/z; daughter ion: 268.1 m/z (quantitative ion), 251.9 m/z (qualitative ion) CE value: 40 V, 55 V; dwell time: 25 ms.

Quality control plasma samples were prepared by mixing multiple plasma samples. The samples were divided into 6 parts, 3 of which were reacted independently according to step 2 (sample pretreatment); the concentrations of the products were measured, and their relative standard deviations (RSDS) were calculated. The other 3 samples of quality control plasma were added to the working solution of sodium sulfide, and the reaction was carried out independently according to step 2 (sample pretreatment). The concentration was determined, and the RSDS was calculated ( Table 2). At the same time, sodium sulfide working solution was added to pure water for three independent reactions to determine its concentration and calculate its RSDS. The RSDS of quality-controlled plasma was 8.77%, the RSDS of quality-controlled plasma was 4.58%, the RSDS of pure water was 8.23%, all of which were less than 20%, and the precision met the requirements of quantitative detection. In addition, the calibration recovery rate was 103.5% according to the above data, namely, (mean value of plasma calibration for quality control‒mean value of plasma calibration for quality control)/mean value of pure water calibration, and the accuracy met the requirements of quantitative detection.

**Table:** **Table 2** Sample concentration and RSDS

Sample	Concentration	RSDS
Quality control plasma sample 1	40612	8.77
Quality control plasma sample 2	38058
Quality control plasma sample 3	45233
Quality control plasma sample marking 1	132537	4.58
Quality control plasma sample marking 2	128728
Quality control plasma sample marking 3	121063
Pure water labelling 1	75779	8.23
Pure water labelling 2	89554
Pure water labelling 3	83975

Quality control plasma samples 1–3 were reacted independently, and quality control plasma samples 1–3 were spiked with sodium sulfide.

In this study, a method for detecting H _2_S in biological samples was constructed. The detection method includes sample pretreatment so that the H _2_S in the sample reacts to generate methylene blue, and then LC-MS is used to detect methylene blue in the sample. The detection value of methylene blue obtained by LC-MS was put into the standard curve to calculate the content of H _2_S corresponding to methylene blue ( Figure 2).

**Figure 2:**
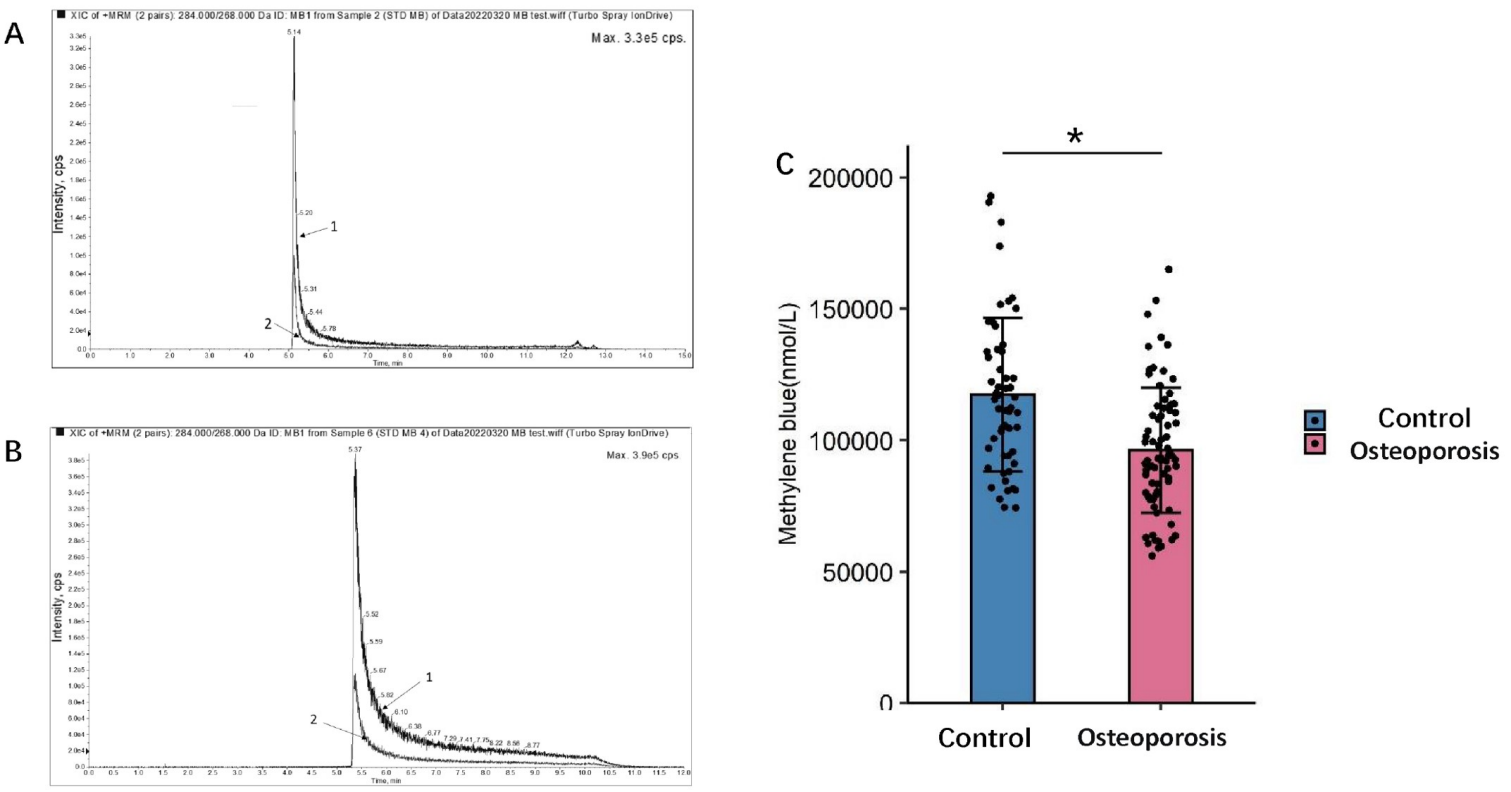
Figure 2 Chromatogram of test samples and standard substances and clinical application (A) The peak chromatogram of methylene blue for the detection of serum samples by the detection method provided in Embodiment 1. (B) The peak chromatogram of methylene blue for the test standard of the test method provided in Embodiment 1. The retention time was 1.47 min. The chromatographic peak pattern of methylene blue was symmetrical and sharp. (C) The serum methylene blue content of osteoporosis patients in the control group. * P<0.05.

The pretreatment included the use of an extraction reagent to react with H _2_S in the sample to obtain a precipitate containing zinc sulfide, which was subsequently reacted with n-dimethyl p-phenylenediamine to generate methylene blue in the presence of neutralizing ferric ions in an acidic medium. In this study, the UHPLC-MRM-MS/MS method was used to detect the target metabolites of H _2_S in samples, and a total of 129 human serum samples were tested. The H _2_S concentration was significantly lower in the postmenopausal osteoporosis patient group than that in the premenopausal female group, and the difference in percent change between the two groups was significant ( Figure 2C).

In mammalian cells, H _2_S is catalyzed by cysteine-gamma-lyase (CSE), cysteine-β synthetase (CBS), cysteine transferase and 3-mercaptopyruvate sulfotransferase (3-MST), that is, endogenous H _2_S, is the main source of H _2_S production *in vivo* [ 9, 10]. In recent years, many studies have shown that H _2_S plays an important regulatory role in bone metabolism, osteoporosis, osteoarthritis and other bone diseases, and may provide a new target for bone metabolism and bone disease research and treatment [3]. In bone formation, H _2_S promotes bone synthesis. H _2_S activates multiple signaling pathways, such as the Wnt signaling pathway, the BMP signaling pathway, the p38-MAPK signaling pathway, and the ERK signaling pathway, through the sulfur hydrogenation of signaling proteins, participating in the regulation of bone formation. H _2_S inhibits inflammation and the antioxidant stress response. In MC3T-E1 osteoblasts, H _2_S reduces the oxidative stress response by inhibiting the MAPK signaling pathway and plays a protective role. Therefore, the demand for H _2_S detection is also increasing in both clinical and scientific research.

In this study, after the reaction of H _2_S to methylene blue was completed, the same sample was tested by LC-MS and spectrophotometry. Both methods have good linearity, while the LC-MS system has a lower quantitation limit. The accuracy of the last point S8 (0.78 μM) of the standard curve in the LC-MS system was 89.43%, which still met the quantitative standard. S8, the last point of the standard curve measured with 195.91% accuracy, was no longer available. In addition, due to the edge effect of the ELISA instrument, the difference between the maximum and minimum values (Δ0.0035) of the three blank parallel measurements was greater than the absorption value (Δ0.0027) of the lower limit S7 (1.56 μM).

The method in this study is proven to be accurate and reliable by verifying its precision and accuracy. During the prevalidation, we evaluated the specificity, reproducibility, matrix effect, and carrier contamination of the methods, which all met the requirements of good reproducibility. In the performance verification, our proposed method’s linear range, recovery rate, imprecision, and dilution consistency are similar to those reported in multiple studies. The linear range was 781.25 nM‒100 μM, and the lower limit of quantitation was 781.25 nM. The linear range was significantly superior to that of the traditional methylene blue spectrophotometer method, and the lower detection limit was 1/12 that of the methylene blue spectrophotometer method, which could meet the detection needs of clinical diagnosis and scientific research. The experimental results of low, medium and high values of imprecision evaluation met the requirements. Compared with Tan *et al* . [11], ^36^S-labelled sulfide dibimane’s method for detecting H _2_S as a medium, although our method has a slightly higher detection limit than theirs, it does not require radioactive elements.

Although the methylene blue spectrophotometer method can meet the requirements of clinical daily detection and easily achieve consistency of imprecision between laboratories, it is still unable to measure serum H _2_S concentrations less than 10 μM. In this study, the AB SCIEX Triple quad 4500+ system was used to establish an LC-MS/MS method for detecting H _2_S; this method has high sensitivity, a high detection level of up to nM, high recovery, and a short detection time. It can be applied in the clinic in the future. However, the mass spectrometry method also has limitations. The instrument is relatively expensive and not widely used in many hospitals. Moreover, there are still several problems to be solved with mass spectrum laboratory information systems. Therefore, it is still challenging to popularize mass spectrometry in the clinic.

## References

[1] Vitvitsky V, Banerjee R. H2S analysis in biological samples using gas chromatography with sulfur chemiluminescence detection.
Methods Enzymol 2015, 554: 111–123. https://doi.org/10.1016/bs.mie.2014.11.013.

[2] Combi Z, Potor L, Nagy P, Sikura KÉ, Ditrói T, Jurányi EP, Galambos K (2023). Hydrogen sulfide as an anti-calcification stratagem in human aortic valve: altered biogenesis and mitochondrial metabolism of H
_2_S lead to H
_2_S deficiency in calcific aortic valve disease. Redox Biol.

[3] Gambari L, Grigolo B, Grassi F (2019). Hydrogen sulfide in bone tissue regeneration and repair: state of the art and new perspectives. Int J Mol Sci.

[4] Olivieri M, Menduni G, Giglio M, Sampaolo A, Patimisco P, Wu H, Dong L (2023). Characterization of H
_2_S QEPAS detection in methane-based gas leaks dispersed into environment. Photoacoustics.

[5] Heshka NE, Hager DB (2015). Measurement of H
_2_S in crude oil and crude oil headspace using multidimensional gas chromatography, deans switching and sulfur-selective detection. J Vis Exp.

[6] Gureev AP, Syromyatnikov MY, Ignatyeva DA, Valuyskikh VV, Solodskikh SA, Panevina AV, Gryaznova MV (2020). Effect of long-term methylene blue treatment on the composition of mouse gut microbiome and its relationship with the cognitive abilities of mice. PLoS One.

[7] Seoane R, Santaeufemia S, Abalde J, Torres E (2022). Efficient removal of methylene blue using living biomass of the microalga chlamydomonas moewusii: kinetics and equilibrium studies. Int J Environ Res Public Health.

[8] Seshadri S, Beiser A, Selhub J, Jacques PF, Rosenberg IH, D′Agostino RB, Wilson PWF (2002). Plasma homocysteine as a risk factor for dementia and Alzheimer’s disease. N Engl J Med.

[9] Yang Y, Wang J, Xiong Z, Yao X, Zhang Y, Ning X, Zhong Y (2021). Prevalence and clinical demography of hyperhomocysteinemia in Han Chinese patients with schizophrenia. Eur Arch Psychiatry Clin Neurosci.

[10] Zheng MH, Li FXZ, Xu F, Lin X, Wang Y, Xu QS, Guo B (2020). The interplay between the renin-angiotensin-aldosterone system and parathyroid hormone. Front Endocrinol.

[11] Tan B, Jin S, Sun J, Gu Z, Sun X, Zhu Y, Huo K (2017). New method for quantification of gasotransmitter hydrogen sulfide in biological matrices by LC-MS/MS. Sci Rep.

